# Drug Repositioning in Glioblastoma: A Pathway Perspective

**DOI:** 10.3389/fphar.2018.00218

**Published:** 2018-03-16

**Authors:** Sze Kiat Tan, Anna Jermakowicz, Adnan K. Mookhtiar, Charles B. Nemeroff, Stephan C. Schürer, Nagi G. Ayad

**Affiliations:** ^1^Department of Psychiatry and Behavioral Sciences, Center for Therapeutic Innovation, Miami Project to Cure Paralysis, Sylvester Comprehensive Cancer Center, University of Miami Brain Tumor Initiative, University of Miami Miller School of Medicine, Miami, FL, United States; ^2^Department of Psychiatry and Behavioral Sciences and Center on Aging, University of Miami Miller School of Medicine, Miami, FL, United States; ^3^Department of Molecular Pharmacology, Center for Computational Sciences, Sylvester Comprehensive Cancer Center, University of Miami Miller School of Medicine, Miami, FL, United States

**Keywords:** glioblastoma, drug repurposing, blood-brain barrier, Library of Integrated Network Based Cell Signatures, LINCS

## Abstract

Glioblastoma multiforme (GBM) is the most malignant primary adult brain tumor. The current standard of care is surgical resection, radiation, and chemotherapy treatment, which extends life in most cases. Unfortunately, tumor recurrence is nearly universal and patients with recurrent glioblastoma typically survive <1 year. Therefore, new therapies and therapeutic combinations need to be developed that can be quickly approved for use in patients. However, in order to gain approval, therapies need to be safe as well as effective. One possible means of attaining rapid approval is repurposing FDA approved compounds for GBM therapy. However, candidate compounds must be able to penetrate the blood-brain barrier (BBB) and therefore a selection process has to be implemented to identify such compounds that can eliminate GBM tumor expansion. We review here psychiatric and non-psychiatric compounds that may be effective in GBM, as well as potential drugs targeting cell death pathways. We also discuss the potential of data-driven computational approaches to identify compounds that induce cell death in GBM cells, enabled by large reference databases such as the Library of Integrated Network Cell Signatures (LINCS). Finally, we argue that identifying pathways dysregulated in GBM in a patient specific manner is essential for effective repurposing in GBM and other gliomas.

## Introduction

Glioblastoma multiforme (GBM) is the most common and aggressive adult primary brain tumor. Despite decades of research and clinical trials, the median survival remains at approximately 14 months. This is in part due to the highly invasive nature of GBM cells, which makes complete surgical resection difficult. In addition, GBM cells develop resistance against the current multimodal treatment regimen that includes the alkylating agent temozolomide (TMZ) and radiation. Furthermore, tumors expressing the DNA repair protein O6-methylguanine methyltransferase (MGMT) are resistant to TMZ. Finally, many targeted therapies fail in clinical trials because they do not effectively cross the blood-brain barrier (BBB). Collectively, these findings necessitate the discovery of novel therapeutic avenues for treating GBM. Impressive technological advances have enabled us to decipher the genetic and cellular makeup of GBM tumors (Verhaak et al., 2010; Clarke et al., 2013). However, the lengthy time required to develop new small molecules and to demonstrate their efficacy and safety in preclinical models is a major impediment for uncovering novel treatments for this devastating disorder.

Drug repositioning may be one means of expediting therapeutic drug development for GBM. Drug repositioning, or drug repurposing, is the method of expanding the therapeutic range of an established Food and Drug Administration (FDA)-approved drug to another disease by identifying a novel use for the drug. One of the reasons drug repositioning may be more advantageous over novel drug discovery is that the pharmacokinetic and safety profiles of that drug are already known. In addition, drug repurposing is considerably less costly and less time intensive than novel small molecule discovery.

The rationale of drug repositioning lies, in part, in the ability of small molecules to target distinct proteins in cells. Different pathways involved in cancer initiation or progression that are considered unrelated to each other can thus be targeted by the same molecule. This concept is also known as “polypharmacology.” This is in contrast to the traditional mindset in drug discovery where the goal is to identify one drug solely for one target, with the hope that this high selectivity can enhance efficacy of the drug and reduce off-target toxicities. In this review, we focus on drug repositioning in glioblastoma, with emphasis on novel uses of psychiatric and non-psychiatric drugs, which are known to cross the BBB. In addition, we highlight recent efforts to utilize systems approaches for identifying repurposing agents in cancer that can be applied to glioblastoma.

One of the most challenging parts of GBM treatment is the complete elimination of the glioblastoma stem cell (GSC) population (Clarke et al., 2013). Among the heterogeneous cellular mass of gliomas, a small subpopulation of cells that are responsible for brain tumor initiation, termed GSCs, have been described to be primarily responsible for the recurrence of malignant glioma, due to their self-renewal ability, and multi-lineage differentiation potential (Jiang et al., 2016; Lubanska and Porter, 2017). These cells recapitulate the parental tumor cells in their complex biological nature. Chemotherapies usually kill the proliferating cells by inducing DNA damage, but do not affect stem cells, which remain at the original site after resection and eventually lead to tumor infiltration and recurrence, as well as GBM resistance to TMZ (Lubanska and Porter, 2017). Hence, many of the recent studies focus on this subpopulation of stem cells. Therefore, in considering repurposing drugs for GBM, it is essential that we consider their efficacy in eliminating GSCs.

## Psychiatric drugs for treating glioblastoma

Several studies have investigated the ability of FDA-approved psychotropic agents to inhibit GBM cell proliferation and migration (Table 1) (Triscott et al., 2012; Lee J. K. et al., 2016). Screens that yielded compounds previously shown to be brain penetrant were considered especially promising because this is an obvious prerequisite for reducing GBM growth in humans. An intact BBB only allows diffusion of lipid-soluble molecules smaller than 400 Da, and molecules which are naturally transported by the existing carrier proteins (Gan et al., 2017). There is also an active efflux mechanism for compounds that enter the brain, mainly due to transporters located at the BBB. Organic anion-transporting polypeptide 1A2 (OATP1A2/SLCO1A2), organic anion transporter 3 (OAT3/SLC22A8), P-glycoprotein (P-gp), multidrug-resistance-associated protein 4 (MRP4/ABCC4), and monocarboxylate transporter 1 (MCT1/SLC16A1) are examples of transporters, which are present at the BBB (Urquhart and Kim, 2009). The restriction of the BBB, which causes low distribution of therapeutic agents in the brain, remains a challenge. Therefore, compounds that have been previously shown to be equally distributed throughout the whole brain are especially attractive for treating GBM patients.

**Table 1:** Summary of primary indications, primary mechanism of actions, antineoplastic mechanism in GBM, and BBB penetrance of different psychiatric and non-psychiatric drugs.

**Drug**	**Mechanism of action**	**Primary indications**	**Mechanism of action in GBM**	**References**	**BBB penetrant**
**PSYCHIATRIC DRUGS**					
**Typical antipsychotics** (*Haloperidol, trifluoperazine, fluphenazine, thioridazine, perphenazine, chlorpromazine*)	Block D_2_ receptors	Psychosis, schizophrenia (mostly positive symptoms), acute mania, bipolar disorders, Tourette syndrome	Suppress proliferation, invasion, and anchorage-independent growth: – de-repressing the IP_3_R, stimulates mass release of Ca^2+^ – inhibiting mitochondrial CcO – inhibiting GPCR σ-receptors – increasing AMPK activity	Kang et al. Oliva et al. Cheng et al. Lee et al.	Yes
**Atypical antipsychotics** (*Olanzapine, clozapine, asenapine, lurasidone, quetiapine, risperidone, aripiprazole*)	Inhibit 5-HT_2A_, D_2_, H_1_, α_1_, and α_2_ receptors	Schizophrenia bipolar disorder, major depressive disorder, generalized anxiety disorder, Huntington's disease	Reduce cell proliferation, anchorage-independent growth, migration, promote apoptosis and necrosis by: – inhibiting the Wnt/β-catenin signaling pathway – downregulating AMPK, c-Jun – cell cycle arrest at G2/M phase – promoting the differentiation into OL-like cells – reducing expression of PIK3CD	Karpel-Massler et al. Guo et al. Ferno et al. Wang et al. Karbownik et al.	Yes
**Tricyclic antidepressants** (*Amitriptyline, imipramine, clopiramine, doxepin, amoxapine*)	Prevent reuptake of norepinephrine and serotonin at the presynaptic receptors	Major depression, neuropathic pain, migraine prophylaxis, anorexia, anxiety disorders	Reduce cell proliferation, cell stemness, limit invasion, induce autophagy, and regulate GSC plasticity and cancer immunity by: – downregulating Sox1, Sox2, Nestin, Ki67, CD44 – inhibiting PI3K/Akt/mTOR signaling pathway – increasing phospho-c-Jun and cytochrome c – regulating NADPH oxidase-mediated ROS generation	Higgins and Pilkington et al. Tzadok et al. Lawrence et al. Bielecka-Wajdman et al. Jeon et al. Levkovitz et al. Munson et al.	Yes
**Selective serotonin receptor inhibitors** (*Sertaline, citalopram, fluoxetine, fluvoxamine, escitalopram, paroxetine*)	Inhibit reuptake of serotonin into neurons	Major depression, bipolar disorder, anxiety disorder	Inhibit GBM invasion, proliferation, increase apoptosis by: – inhibiting actin polymerization – lamellipodia suppression – decreasing FAK, Akt and mTOR phosphorylation – activating AMPA receptors, increases Ca^2+^ influx into mitochondria – releasing cytochrome c, caspase-9, caspase-3, and PARP, triggering apoptosis	Levkovitz et al. Hayashi et al. Munson et al. Tzadok et al. Liu et al.	Yes
**Sedative hypnotics** (*Benzodiazepines: Diazepam, lorazepam, triazolam, temazepam, oxazepam, midazolam*)	Facilitate GABA_A_ receptor complex action in CNS by increasing the frequency of Cl^−^ channel opening	Anxiety disorders, spasticity, status epilepticus, detoxification, night terrors, sleepwalking	Inhibit cell proliferation, sensitize GBM cells to chemotherapy by: – inactivating Rb protein at G1/S transition (cell cycle arrest) – facilitating hypericin-induced and etoposide-induced cytotoxicity	Chen et al. Sarissky et al.	Yes
**Antiepileptics** (*Sodium valproate, carbamazepine, levetiracetam*)	Prolong Na^+^ channel inactivation and inhibits GABA transaminase	Myoclonic seizures, migraines, bipolar disorders	Reduce cell proliferation, increase autophagy, increase GBM sensitivity to TMZ and radiosensitivity by: – inhibiting HDAC – downregulating MGMT – enhancing p53 expression – increasing binding of HDAC1/mSin3A complex to the MGMT promoter – binding to SV2A, enhance GABA release	Zhang et al. Chinnaiyan et al. Tseng et al. Bobustuc et al. Lee et al. Pinheiro et al. Knudson-Baas et al. Peddi et al.	Yes
**Disulfiram**	Inhibits ALDH enzyme	Alcohol abuse	Inhibits proliferation, self-renewal, increases sensitivity by: – inhibiting ALDH – inhibiting proteasome and NF-κB pathways by chelation – inhibiting p97 pathway – decreasing MGMT expression, PLK1 protein and mRNA	Triscott et al. Chen et al. Choi et al. Paranjpe et al. Lun et al. Skrott et al.	Yes
**NON-PSYCHIATRIC DRUGS**					
**Mebendazole**	Microtubule inhibitor	Antihelmintic drug	– Inhibiting microtubule polymerization – Inhibiting protein kinase – Inducing metaphase arrest	De Witt et al.	Polymorph A – No; Polymorph B and C - Yes
**Vincristine**	Microtubule inhibitor	Chemotherapy in various cancers	Prevents mitotic spindle formation and M-phase arrest	De Witt et al. Kipper et al.	No
**Clomifene**	Antagonist at estrogen receptors in the hypothalamus	Women infertility due to anovulation, PCOS	– Inhibiting mutant IDH1, reduces D-2HG – Increasing apoptosis	Zheng et al. Yaz et al.	Unknown
**Biguanides** (*Metformin, phenformin*)	Inhibit gluconeogenesis, increase glycolysis, and increase insulin sensitivity by promoting peripheral glucose uptake	Type II diabetes mellitus	Inhibit cell proliferation, migration, angiogenesis, TMZ resistance, self-renewal, stemness of GSC and induce apoptosis by: – inhibiting complex I of the ETC and mTORC1 – inhibiting AMPK and STAT3 pathways – increases of miR-124 and let-7 – inhibiting glutamate dehydrogenase, reduces D-2HG – inhibiting CLIC1, leads to G1 arrest	Jiang et al. Molenaar et al. Ferla et al. Ucbek et al. Yang et al. Elmaci et al. Kast et al. Aldea et al. Lee et al. Gritti et al.	Yes
**Repaglinides**	Insulin secretagogue	Type II diabetes mellitus	Inhibit proliferation, migration, and increase immune cytotoxicity by: – inducing apoptosis – suppressing autophagy – downregulating Bcl-2, Beclin-1 and PD-L1	Xiao et al.	Modest
**Cyclin-Dependent Kinases** (***First generations**: Flavopiridol; **Second generation**: Palbociclib, dinacliclib, roscovitine, milciclib, purvalanol A*)	Cell cycle checkpoint regulators	Anti-cancer drugs	Cytotoxic to cells, reduce cell proliferation by: – inhibiting DNA repair activity at G2M transition – inactivating Rb1 phosphorylation	Jane et al.	Varies (Yes for abemaciclib and pablociclib)
**EGFR Inhibitors** (***TKI**: erlotinib, gefitinib; **mAbs**: nimotuzumab, cetuxima*b)	Prevent ligand binding, and tyrosine kinase activation	Anti-cancer drugs (NSCLC, HNSCC)	Tyrosine kinase inhibitors	Miranda et al.	Varies (Low)
**Statins** (*Lovastatin, pravastatin, rosuvastatin, simvastatin*)	HMG-CoA reductase inhibitors	Lipid lowering agents	Cytotoxic to GBM cells by: – TRAIL-sensitizing effect – increasing Bim – reducing MAPKs-dependent pathway activations with GTPase activation; – suppression of ERK1/2 and Ras/PI3K/Akt pathway – Activation of JNK1/2	Yanae et al. Tapia-Perez et al.	Varies

^*^ALDH, acetaldehyde dehydrogenase; AMPA, α-amino-3-hydroxy-5-methyl-4-isoxazolepropionic acid; AMPK, 5′-AMP-activated protein kinase; BBB, blood brain barrier; Bcl-2, B-cell lymphoma 2; CcO, cytochrome c oxidase; CLIC1, chloride intracellular channel-1; CNS, central nervous system; D_2_, dopamine 2; D-2HG, D-2-hydroxyglutaricacid; ERK1/2, extracellular regulated kinase 1/2; ETC, electron transport chain; FAK, focal adhesion kinase; GABA_A_, γ aminobutyric acid A; GBM, glioblastoma multiforme; GPCR, G-protein coupled receptor; GSC, glioma stem cell; HDAC, histone deacetylase; HMG-CoA, β-hydroxy-β-methylglutaryl coenzyme A; HNSCC, squamous cell carcinoma of head and neck; IDH, isocitrate dehydrogenases; IP_3_R, inositol 1,4,5-triphosphate receptor; JNK1/2, c-Jun N-terminal kinase $\frac{1}{2}$; mAbs, monoclonal antibodies; MAPKs, mitogen-activated protein kinases; MGMT, O6-methylguanine-DNA methyltransferase; mTORC1, mammalian target of rapamycin complex 1; NSCLC, non-small-cell lung carcinoma; OL, oligodendrocyte; PARP, poly (ADP-ribose) polymerase; PCOS, polycystic ovarian syndrome; PD-L1, programmed death-ligand 1; PIK3CD, phosphatidylinositol-4,5-bisphosphate 3-kinase catalytic subunit delta; PLK1, serine/threonine-protein kinase; Rb, retinoblastoma; ROS, reactive oxygen species; STAT3, signal transducer and activator of transcription 3; SV2A, synaptic vesicle glycoprotein 2A; TKI, tyrosine kinase inhibitors; TMZ, temozolamide; TRAIL, TNF-related apoptosis-inducing ligand.

Importantly, many GBM patients are treated with psychopharmacological agents because they suffer from comorbid psychiatric disorders such as anxiety, depression with suicidal ideation, psychosis, and acute confusional status (Lee J. K. et al., 2016). Interestingly, the incidence of cancer occurrence is inversely proportional to antipsychotic drug treatment in patients with schizophrenia, perhaps suggesting that there is a benefit to treatment with antipsychotic drugs for cancer patients (Barak et al., 2005; Tran et al., 2009). Whether this is due to a direct effect on tumor growth or indirectly due to the psychological benefits for the medications remains to be determined. Although many of the primary targets of these drugs are present in GBM cells, most reports discuss the off-targets effects of these drugs and favor polypharmacology. Below we discuss some drugs commonly used in psychiatry that have potent anti-proliferative properties *in vitro* and *in vivo*.

### Typical antipsychotics (neuroleptics)

Antipsychotic drugs, also known as neuroleptics or major tranquilizers, are primarily used in the treatment of psychosis, schizophrenia, acute mania, bipolar disorder, and Tourette syndrome. Haloperidol, trifluoperazine, fluphenazine, thioridazine, perphenazine, and chlorpromazine (CPZ) are some of the commonly used antipsychotics. All typical antipsychotics block dopamine D_2_ receptors.

**Figure 1:**
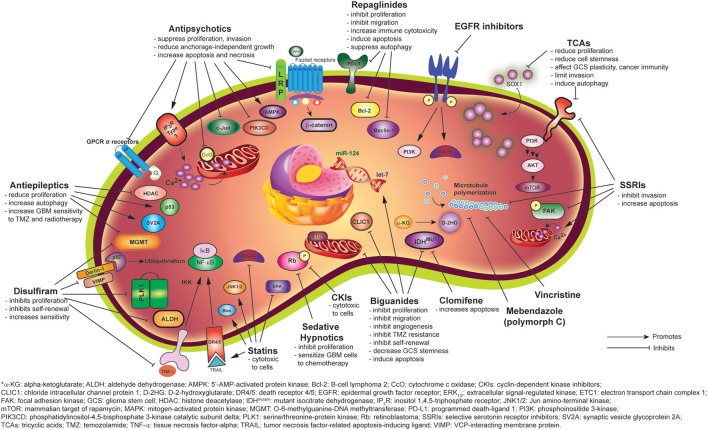
Mechanisms of anti-gliomagenic effects of different psychiatric and non-psychiatric drugs as demonstrated via *in vitro* studies.

Antipsychotics suppress proliferation, invasion, and anchorage-independent growth of GBM cells (Oliva et al., 2017; Pinheiro T. et al., 2017). Dopamine receptor subtype 2 is present in GBM cells and is responsible for the mitogenic signaling (Bartek and Hodny, 2014). There have been several mechanisms of action proposed for their potential anti-tumor effect (Table 1). For example, a recent publication by Kang et al. showed that trifluoperazine binds to a Ca^2+^-binding protein, calmodulin subtype 2 (CaM2), de-represses the Ca^2+^ release channel inositol 1,4,5-triphosphate receptor (IP_3_R) subtype 3, and subsequently stimulates the irreversible mass release of Ca^2+^ in GBM cells (Kang et al., 2017) (Figure 1). Ca^2+^ is essential for numerous functions in cells such as regulating gene expression and metabolism (Kang et al., 2017). Intracellular Ca^2+^ homeostasis is tightly regulated in a biological system and an alteration in Ca^2+^ levels can result in cell death (Kang et al., 2017). A phenothiazine, CPZ, was demonstrated by Oliva et al. to inhibit mitochondrial cytochrome c oxidase (CcO) in chemoresistant glioma cells and GSCs when CcO subunit 4 isoform 1 (COX4-1) is present, but not COX4-2 (Oliva et al., 2017). However, this was only true for TMZ-resistant cells as TMZ-sensitive cells were not affected by CPZ. Attenuated CcO reduces the efficacy of mitochondrial OxPhos dependent ATP-linked respiration and lowers reactive oxygen species production, thereby lowering glioma progression (Oliva et al., 2017). COX4 affects the sensitivity of GBM cells to CPZ (Oliva et al., 2017). Increased CcO activity and increased COX4-1 expression were observed to be associated with worse prognosis in GBM (Oliva et al., 2017). Oliva et al. also demonstrated the beneficial effect of CPZ in prolonging survival in an *in vivo* preclinical study (Oliva et al., 2017). Importantly, there were no adverse behavioral effects noticed with CPZ use in this model, suggesting that similar use in GBM patients could be well-tolerated.

Another potential means for reducing GBM growth is via inhibiting G-protein coupled receptors (GPCRs) that regulate GBM cell proliferation. For instance, pimozide inhibits σ-receptors, one of the atypical GPCRs expressed, and thus attenuates GBM proliferation (Lee J. K. et al., 2016) (Figure 1). Another antipsychotic, thioridazine, has been shown to be cytotoxic to GBM cells by increasing 5′-AMP-activated protein kinase (AMPK) activity, which is downstream of GPCR signaling (Cheng et al., 2015). Given the substantial literature associated with GPCR signaling, it will be important to correlate clinical efficacy of drugs affecting GBM progression in clinical trials with the signaling pathway the drug affects.

### Atypical antipsychotics

Although the exact molecular mechanisms underlying the therapeutic actions of atypical antipsychotics remain obscure, they have multiple effects on dopamine, 5-HT_2_, α-, and H_1_-receptors. Their low-risk profiles make them especially attractive for repurposing in GBM. Olanzapine, clozapine, asenapine, lurasidone, quetiapine, risperidone, aripiprazole, brexpiprazole, and ziprasidone are some examples of second-generation atypical antipsychotics (Table 1).

Olanzapine is used in the treatment of schizophrenia, bipolar disorder, and neurological conditions such as Huntington's disease. Olanzapine is an antagonist of the serotonin (5-HT_2A_) and dopamine (D_2_) receptors. In the cancer field, olanzapine has been used to control pain and chemotherapy associated nausea. Olanzapine has emerged as an attractive therapeutic candidate for repurposing in brain cancer as it reduced glioblastoma cell expansion *in vitro* and *in vivo* (Karpel-Massler et al., 2015; Karbownik et al., 2016). Karpel-Massler et al. reported that olanzapine has antineoplastic capability and its cytotoxicity effect *in vitro* is enhanced when combined with TMZ (Karpel-Massler et al., 2015). Furthermore, olanzapine reduces GBM cancer cell proliferation, decreases anchorage-independent colony formation, inhibits migration, and induces mixed apoptosis and necrosis in GBM cells (Karpel-Massler et al., 2015). The mechanism of action includes downregulation of the Wnt/β-catenin pathway and c-Jun and is also thought to be dependent on the extracellular concentration of phospholipase D and other factors (Karpel-Massler et al., 2015) (Figure 1). However, the efficacy of olanzapine likely varies among different GBM cell lines given the heterogeneous nature of GBM as another study found that treatment of glioma cells with olanzapine did not affect viability (Ferno et al., 2006). The increase in AMPK thought to promote cell death in the Karpel-Masler study was not observed in this study (Karpel-Massler et al., 2015). Another possibility to account for the differential effects is that the concentrations used were vastly different. Indeed, the concentrations used in the Karpel-Massler study were very high and not likely to be attained in the clinical setting.

Another atypical antipsychotic, quetiapine, acts as an antagonist at serotonin (5-HT_1A_ and 5-HT_2A_), dopamine (D_1_ and D_2_), histamine (H_1_), and adrenergic (α_1_ and α_2_) receptors. It is an FDA-approved drug for the treatment of schizophrenia, bipolar disorder, and often used in major depressive disorder (MDD) as well as generalized anxiety disorder. In a recent publication, Wang et al., demonstrated that quetiapine suppresses GBM cell growth *in vitro* and *in vivo* (Wang et al., 2017b). High doses of quetiapine suppress GSC proliferation by arresting cells at the G2/M phases of the cell cycle. Importantly, quetiapine improves survival of mice bearing glioma. The proposed mechanism of action involves promoting the differentiation of GSCs into oligodendrocyte (OL)-like cells by inhibiting the Wnt/β-catenin signaling pathway (Wang et al., 2017b) (Figure 1). This anti-gliomagenic property is attributed to the finding that well-differentiated cells are more sensitive to chemotherapy than less differentiated ones (Persson et al., 2010). This is significant in demonstrating that quetiapine may inhibit TMZ-resistant glioma (Wang et al., 2017b).

Another mechanism through which quetiapine controls cell growth is via downregulation of the phosphoionositide 3-kinase (PI3K) pathway, a major driver of GBM cell proliferation (Figure 1). Karbownik et al. in 2016 demonstrated that quetiapine reduces mRNA expression of the PI3K component PIK3CD in GBM cells (Karbownik et al., 2016).

### Tricyclic antidepressants

Approximately 90% of GBM patients are reported to have significant depressive symptoms and neurological disturbances (Bielecka-Wajdman et al., 2017). One can easily foresee that the severe stress associated with GBM patients both from the poor prognosis and the consequences of therapy contribute to depression. Furthermore, oncologists often also prescribe these antidepressants to control chronic and neuropathic pain, migraines, anorexia, anxiety disorders, and circadian rhythms. Tricyclic antidepressants (TCAs) such as amitriptyline, imipramine, clopiramine, doxepin, and amoxapine act primarily by preventing the reuptake of norepinephrine and serotonin at presynaptic terminals (Higgins and Pilkington, 2010).

Although the antidepressant effects of TCAs are well established, the anticancer effect remains a big question due to many conflicting reports discussing whether antidepressants can induce or reduce tumorigenesis. TCAs reduce cell proliferation in rat C6 glioma, human neuroblastoma, and human astrocytoma cells (Higgins and Pilkington, 2010; Tzadok et al., 2010; Lawrence et al., 2016). Bielecka-Wajdman et al. also demonstrated that antidepressants, especially amitriptyline and imipramine, can reduce the “stemness” of the GSCs at a rate dependent on the oxygen content of the hypoxic microenvironment in which the tumor resides. The low oxygen content in tumors is a common issue for most cancers and it is responsible for GBM invasion (Monteiro et al., 2017). Bielecka-Wajdman et al. observed a downregulation of the “stemness genes” Sox1, Sox2, Nestin, Ki67, and CD44 after TCA treatment (Bielecka-Wajdman et al., 2017). They also hypothesized that TCAs can affect GCS plasticity and cancer immunity by regulating pro- and anti-inflammatory cytokines, immune cells, and reactive oxygen species (Bielecka-Wajdman et al., 2017). Hence, it is possible that TCA can trigger the host immune response.

In addition, imipramine, as demonstrated by Jeon et al., reduces U-87MG glioma cell growth by inhibiting the PI3K/Akt/mTOR signaling pathway and inducing autophagy, but not apoptosis (Jeon et al., 2011) (Figure 1). This is in response to the report from Levkovitz et al. where they demonstrated that some TCAs but not imipramine, increase apoptosis (Levkovitz et al., 2005). They observed a rapid rise in phospho-c-Jun levels and increased mitochondrial cytochrome c release in glioma cells after TCA treatment (Levkovitz et al., 2005). Munson et al. in 2012 also demonstrated that imipramine blue is able to limit the invasion of GBM cells and this “containment” helps to enhance the effect of chemotherapy at the tumor field. This is suggested to be modulated by NADPH oxidase-mediated reactive oxygen species generation (Munson et al., 2012). These off-targets effects seen with TCA use are likely to inhibit GBM cells survival.

A recent nation-wide study conducted by Pottegard et al. in Denmark investigated the protective effects of TCAs against gliomas. In a sample of 75,340 control patients and 3,767 patients with glioma, long term (>3 years) use of TCAs was inversely correlated with glioma risk, with an odds ratio of 0.72 (Pottegard et al., 2016). This is contrary to the observation reported by Walker in 2012 in the United Kingdom that exposure to TCA post-diagnosis of glioma does not improve survival (Walker et al., 2012). However, patients in this study were not receiving TCAs previously, so the experimental designs in these two studies are quite different.

### Selective serotonin reuptake inhibitors

Selective serotonin reuptake inhibitors (SSRIs), the most commonly used antidepressants, increase serotonin concentrations at the synapses and activate the postsynaptic neurons (Table 1). Commonly used SSRIs are sertraline, citalopram, fluoxetine, fluvoxamine, escitalopram, and paroxetine. A report from the Glioma Outcomes Project has shown that patients who are depressed, either during the surgery or post-operative management period, are more likely to suffer from more comorbidities and have an increased rate of death (Caudill et al., 2011). Although Caudill in 2010 concluded that the use of SSRIs is safe in GBM patients, there is still a debate as they are associated with a higher risk of seizures (Caudill et al., 2011). Compared to citalopram and sertraline, fluoxetine, and paroxetine can inhibit the CYP450 2D6 isoenzyme and lead to drug-drug interactions that may account for the higher toxicity of these medications in GBM patients (Rooney and Grant, 2012).

SSRIs have recently gained much attention for potential anti-GBM property due to their BBB penetration properties and favorable safety profile. Fluvoxamine, which is able to be selectively transported into the brain at higher concentration without causing peripheral side effects, is safe to be used in treating depression in GBM patients. In addition to reducing cell proliferation, SSRIs can induce apoptosis in GBM (Levkovitz et al., 2005). Fluvoxamine has been demonstrated to reduce actin polymerization through inhibition of actin polymerization-related proteins, thereby reducing GBM cell invasion via lamellipodia suppression (Hayashi et al., 2016) (Figure 1). Hayashi et al. demonstrated that fluvoxamine decreases FAK phosphorylation at Y397, Akt phosphorylation at T308 and S473, as well as mTOR phosphorylation at S2448 and S2481 (Hayashi et al., 2016) (Figure 1). Although fluvoxamine does not affect GBM cell proliferation, a reduction in proliferating cells was seen in fluvoxamine treated tumors, and fluvoxamine was shown to reduce tumor burden in mice bearing tumors from human glioma-initiating cells, suggesting that the positive effects of fluvoxamine in preclinical glioma models is achieved via reducing the invasive capacity of tumor cells. In their model, fluvoxamine maleate is administered at 50 mg/kg/day, which is higher than the daily human equivalent dose that is usually given to patients in the clinic. (Hayashi et al., 2016).

In contrast to fluvoxamine, fluoxetine induces glioma cell death. Importantly, fluoxetine is not toxic to primary astrocytes and neurons (Liu et al., 2015). Fluoxetine directly binds to GluR1, activates AMPA receptors and increases Ca^2+^ influx into the mitochondria (Liu et al., 2015). This Ca^2+^ influx subsequently induces mitochondrial membrane damage and releases cytochrome c, as well as activating caspase-9, caspase-3, and poly (ADP-ribose) polymerase (PARP), thereby triggering apoptosis (Liu et al., 2015). Interestingly, fluoxetine is similar to TMZ in reducing GBM growth *in vivo* (Munson et al., 2012). Tzadok et al. in 2010 also demonstrated the synergistic effect of fluoxetine, sertraline, or perphenazine with the tyrosine kinase inhibitor imatinib in reducing GBM cell proliferation, suggesting a potential use for GBM therapy (Tzadok et al., 2010).

### Sedative hypnotics

Benzodiazepines are often used as a general anesthetic and are also indicated for patients suffering from several anxiety disorders, spasticity, status epilepticus, detoxification, night terrors, and sleepwalking. Diazepam, lorazepam, triazolam, temazepam, oxazepam, and midazolam are the most widely available benzodiazepines (Table 1). They facilitate γ aminobutyric acid A (GABA_A_) receptor complex action in the central nervous system by increasing the frequency of Cl^−^ channel opening (Chen et al., 2013). In GBM patients, diazepam can help alleviate post-cancer therapy anxiety and inhibit chemotherapy-associated delayed emesis. Even though benzodiazepines easily cross the BBB, the need for a higher dose to achieve efficacy in anticancer therapy remains a safety concern.

In 2013, Chen et al. investigated the anti-proliferative property of diazepam in human glioblastoma (Chen et al., 2013). They demonstrated that by inactivating the cell cycle protein Rb, diazepam can cause a cell cycle arrest at the G0/G1 phase in human GBM cells in a dose-dependent manner (Figure 1). This result adds on to an earlier study, which showed that diazepam facilitates hypericin-induced and etoposide-induced cytotoxicity in GBM cells (Sarissky et al., 2005). Altogether, this demonstrates that diazepam not only sensitizes GBM cells to chemotherapy, but also kills tumor cells.

### Antiepileptics/anticonvulsants

Nearly 22–60% of GBM patients exhibit epileptic seizures as an initial clinical complication (Van Nifterik et al., 2012). Sodium valproate or valproic acid (VPA) is one of the most commonly prescribed antiepileptic drugs (AEDs) that has been used for decades. The prescription is usually justified after a first and single seizure in a GBM patient. VPA is also utilized for the treatment of myoclonic seizures, migraines and bipolar disorder. Sodium valproate prolongs Na^+^ channel inactivation and inhibits gamma-butyric acid (GABA) transaminase, hence increasing the concentration of GABA (Table 1).

Sodium valproate or VPA is also a histone deacetylases (HDACs) inhibitor that has been proposed as a potential adjuvant in cancer treatment. Histone lysine residue acetylation and deacetylation are among the most widely characterized posttranslational modifications in epigenetics. Histone deacetylases promote neoplasia by condensing chromatin and repressing transcription of tumor suppressor genes, and these HDACs are often overexpressed in GBM (Rundle-Thiele et al., 2016). As epigenetic modifiers, HDAC inhibitors can increase the cancer cell's sensitivity to ionizing radiation, while preventing normal cells from being killed by radiotherapy (Zhang et al., 2016). Sodium valproate exposure increases histone hyperacetylation in glioma cells, inhibits cell growth, and increases cell radiosensitivity (Chinnaiyan et al., 2008; Van Nifterik et al., 2012; Lee C. Y. et al., 2016; Zhang et al., 2016; Tseng et al., 2017). Sodium valproate has also been used in combination with other chemotherapies such as TMZ, topoisomerase inhibitors, and carboplatin in medulloblastoma and glioma studies (Felix et al., 2011). By contrast, Lee et al. demonstrated that at a therapeutic dose, sodium valproate alone inhibits <20% of cell proliferation (Lee C. Y. et al., 2016). However, when combined with TMZ, VPA shows a significant antineoplastic impact in TMZ-resistant glioma cells via downregulating MGMT expression (Figure 1). This combination therapy also showed promising results in a Phase II clinical trial of newly diagnosed GBM patients. Interestingly, a hybrid compound of TMZ and an HDAC inhibitor named HYBCOM was developed in order to minimize resistance (Pinheiro R. et al., 2017). The authors were able to demonstrate the efficacy of this compound in reducing glioma cell proliferation through selective autophagy in tumor cells and reduced multi-drug resistance, as compared to TMZ alone. However, VPA can inhibit several enzymes, such as CYP2C coenzymes, epoxide hydroxylase, and uridine diphosphate-glucuronosyltransferase, which may be linked to its unfavorable side effects. Killick-Cole et al. also proposed repurposing VPA for the treatment of diffuse intrinsic pontine glioma (DIPG), a deadly pediatric brain tumor (Killick-Cole et al., 2017). In DIPG, sodium valproate reduced survival of *ex vivo* DIPG cells, induced apoptosis, and showed minimal toxicity to rat hippocampal neuronal and glial cells. In addition, pre-conditioning of DIPG cells by sodium valproate is synergistic with carboplastin in inducing cytotoxicity in these cells (Killick-Cole et al., 2017). However, there is no report on targeting Na^+^ in GBM by an antiepileptic.

Importantly, some of the drugs or drug combination can influence the recognition of GBM cells by the immune system. Hence it is possible that these drugs can also activate a host immune response. For instance, the alkylating agent administered to GBM patients, TMZ, can stimulate the expression of stress-induced antigens such as NKG2D ligands (MICA and ULBPs) on GBM cells (Chitadze et al., 2016). This sensitizes them to be killed by anti-tumor effector cells. Interestingly, the same effect is also seen in GBM cells when treated with HDAC inhibitors (Adamopoulou and Naumann, 2013).

Recently, nation-wide based data from 1,263 GBM patients in Norway from 2004 to 2010 showed that the choice of AED does not affect survival of GBM patients (Knudsen-Baas et al., 2016). Happold and colleagues in 2016 prospectively analyzed a pooled dataset of 1,869 newly diagnosed GBM patients recruited from four different clinical trials and showed that the use of sodium valproate does not correlate with survival (Happold et al., 2016).

By contrast, carbamazepine was the most frequently prescribed AED for GBM patients from 2004 to 2006 in Norway. However, this shifted toward levetiracetam (LEV) at a later period, namely from 2009 to 2010. LEV, another commonly used anticonvulsant, is effective in treating and preventing focal seizures, which are common in patients with intracranial tumors. It binds to the vesicular protein SV2A and enhances the release of GABA. It also penetrates the BBB rapidly and has a high therapeutic index, as compared to other AEDs (Knudsen-Baas et al., 2016). Importantly, this drug may be especially promising in treating GBM because of its lack of interaction with chemotherapy agents.

Peddi et al. in 2016 reported the first possible case of glioblastoma regression after combination treatment of dexamethasone and LEV intended for seizure prophylaxis (Peddi et al., 2016). This opens up many questions regarding the cause of this remarkable finding. LEV, as demonstrated by Bobustuc et al., has the ability to abrogate glioma cell proliferation and increase GBM cellular sensitivity to TMZ (Bobustuc et al., 2010). LEV enhances the expression of the tumor suppressor protein p53 and increases binding of the HDAC1/mSin3A complex to the MGMT promoter (Bobustuc et al., 2010). This survival benefit is further validated in a prospective randomized study by Kim et al. in 2015, showing that the median progression-free survival (PFS), and overall survival (OS) for GBM patients taking LEV in combination with TMZ is significantly longer than those receiving TMZ alone (Kim et al., 2015).

### Disulfiram

Disulfiram (tetraethylthiuram disulfide), which is currently used to treat alcoholism, is one of the most promising FDA-approved drugs for repurposing in GBM. Disulfiram was initially discovered in the rubber manufacturing industry in 1937, where it was observed that rubber workers who were exposed to disulfiram developed flu-like symptoms whenever they imbibed alcohol (Triscott et al., 2015). Based on this serendipitous finding, disulfiram has been utilized for the treatment of alcohol abuse and has been used for more than 60 years. The mechanism of action for disulfiram is that it inhibits the liver acetaldehyde dehydrogenase (ALDH) enzyme, which normally catalyzes the oxidation of acetaldehyde to acetate with the aid of the NAD^+^ cofactor (Triscott et al., 2012). After ALDH inhibition, acetaldehyde accumulates, which contributes to flushing, sweating, headache, nausea, and other hangover symptoms.

Multiple *in vitro* and *in vivo* studies have indicated that disulfiram may be effective for the treatment of brain tumors. Triscott et al. in 2012 demonstrated that even low-dose disulfiram inhibits proliferation and self-renewal of GBM cells that are resistant to TMZ, without affecting normal human astrocytes (Triscott et al., 2012). However, the dose of disulfiram used in their study is higher than the 250 mg/day dose given to patients, and therefore the potential utility is questionable (Triscott et al., 2012). In 2015, the same group also demonstrated that GBM cells are sensitive to disulfiram, but not TMZ (Triscott et al., 2015). Choi et al. showed that disulfiram crosses the BBB in mice and inhibits atypical teratoid rhabdoid tumors (Choi et al., 2015). Paranjpe et al. reported that disulfiram can increase cell killing by decreasing MGMT expression in xenograft models (Paranjpe et al., 2014). Collectively, these studies suggest that disulfiram should be considered for the treatment of GBM.

The antineoplastic property of disulfiram may be due to several mechanisms. As the most established pathway affected by disulfiram is the ALDH enzyme, ALDH has been shown to be upregulated in tumor cells with enhanced tumor growth in xenografts as well as resistance to chemotherapies (Triscott et al., 2015) (Figure 1). Furthermore, disulfiram inhibits the proteasome and NF-κB pathways (Triscott et al., 2015). Disulfiram is pharmacokinetically converted into a smaller metabolite called diethyldithiocarbamate (DTC), which chelates with copper or zinc ions (Chen et al., 2006; Lun et al., 2016). The complexes formed can suppress proteasome activity and increase oxygen free radicals. Indeed, some studies have shown that combining disulfiram with copper can increase cytotoxicity in cancer cells *in vitro* and *in vivo*. A very recent study demonstrated reduction of tumor volume in mice treated with disulfiram with copper gluconate as compared to disulfiram alone. The chelation product after disulfiram ingestion, the DTC-copper complex, was hypothesized to bind NPL4, and subsequently inhibit the p97 pathway, thus resulting in cell death (Skrott et al., 2017) (Figure 1). However, a similar effect was observed in normal cells, suggesting that identifying a therapeutic window is essential when using disulfiram. In addition, Choi et al. cautioned that there are potential side-effects when ingesting too much copper and zinc, again suggesting that a careful dosing strategy is needed when using disulfiram and copper and zinc (Choi et al., 2015).

Disulfiram inhibits cancer stem cells in lung cancer, breast cancer, ovarian cancer, pancreatic cancer, and blood cancers. Importantly, disulfiram is able to penetrate the BBB, favoring its use in treating brain tumors. Despite having an already established safe therapeutic index, many ongoing studies are investigating a dosing schedule and chemotherapy combination that will deliver the maximum effects in tumor cells (Triscott et al., 2012). These findings suggest the potential role of disulfiram to be repurposed for use in GBM, and potentially in pediatric brain tumors in the future.

## Non-psychiatric drugs

In addition to drugs used in psychiatry, which have favorable brain exposures for treating GBM and other brain cancers, several groups have focused on repurposing FDA approved compounds not used in psychiatry. However, the challenge here is to determine the efficacy of these compounds in reducing tumor growth in the brain. For many of these compounds, the brain exposure profiles and the pharmacokinetics properties have not been determined. Therefore, considerable efforts are needed to determine whether combination therapies of these repurposed compounds with the current standard-of-care will either facilitate or inhibit BBB penetrance of these compounds.

### Mebendazole

A microtubule inhibitor, mebendazole is an FDA-approved antihelmintic drug. The ability of mebendazole to form different polymorphs (A, B, and C) depends on the crystallization conditions. Polymorph A does not penetrate the BBB as efficiently as polymorphs B and C (Table 1) (Bai et al., 2015). Mebendazole generally has a benign safety profile, although it has been shown to cause bone marrow suppression and liver toxicity at higher doses (De Witt et al., 2017).

Mebendazole can exhibit anti-tumor effects by inhibiting protein kinases. It is unknown whether the cell death observed in tumor cells treated with mebendazole is also mediated by the microtubule destabilizing effect. It was demonstrated by De Witt et al. that mebendazole inhibits microtubule polymerization and induces metaphase arrest, very similar to the mechanism of action of another microtubule inhibitor, vincristine (De Witt et al., 2017) (Figure 1). Furthermore, mebendazole polymorph C shows a survival benefit in a model of C57BL/6 mice bearing GL261 cells (Bai et al., 2011, 2015; De Witt et al., 2017). Although the authors observed increased survival with 100 mg/kg of administered mebendazole as compared to 50 mg/kg, it is close to the maximum tolerated dose and may not be achievable in humans. However, due to the efficacy of mebendazole in tumor suppression, it was recommended that mebendazole replaces vincristine in neuro-oncology management.

### Vincristine

From the family of vinca alkaloids, vincristine binds to β-tubulin, and inhibits microtubule polymerization, thereby inhibiting the formation of the mitotic spindle during cell division (M-phase arrest) (De Witt et al., 2017) (Figure 1).

In contrast to mebendazole, vincristine cannot penetrate the BBB well due to its large molecular size (825 Da) and its tendency to be transported (De Witt et al., 2017). Vincristine is currently used in the treatment of 1p/19q co-deleted anaplastic oligodendroglioma and low-grade glioma when combined with procarbazine and lomustine (CCNU) (De Witt et al., 2017). Although vincristine has been used in brain tumor management, the poor BBB penetrance and its significant side effects remain the biggest concern. Importantly, De Witt et al. demonstrated that at the same dose as mebendazole, vincristine failed to improve survival *in vivo* (De Witt et al., 2017).

The beneficial effect of a combination of two microtubule inhibitors in patients is questionable. Although the combination of vinblastine and mebendazole was shown to improve the sensitivity of resistant glioma cells to TMZ (Kipper et al., 2017), mebendazole and vincristine co-administration exacerbates peripheral neuropathy side effects *in vivo*. Hence, more research needs to be done to determine how to utilize similar combination therapies while minimizing toxicities.

### Clomifene

Clomifene is commonly used as a selective estrogen receptor modulator in the treatment of female infertility due to anovulation, such as polycystic ovarian syndrome (PCOS) (Zheng et al., 2017). It is also used off-label for treating hypogonadism in men (Zheng et al., 2017). It acts as an antagonist at estrogen receptors in the hypothalamus and thus prevents normal feedback inhibition, which subsequently increases release of Luteinizing Hormone (LH) and Follicle-Stimulating Hormone (FSH) from the anterior pituitary gland, leading to ovulation.

By utilizing structure-based virtual ligand screening, Zheng et al. identified clomifene as an inhibitor of mutant isocitrate dehydrogenases (IDH) 1, which is essential for tumorigenesis in multiple cancers (Zheng et al., 2017). They demonstrated that mutant IDH1 was selectively inhibited by clomifene, thus reducing the accumulation of downstream D-2-hydroxyglutaricacid (D-2HG) (Zheng et al., 2017) (Figure 1). D-2HG drives carcinogenesis by inhibiting histone demethylases and this increases global methylation of histones and DNA (Zheng et al., 2017). The administration of clomifene also increases apoptosis of glioma cancer cells with IDH1 mutations *in vitro* and *in vivo*, without causing any side effects of hepatotoxicity or nephrotoxicity (Zheng et al., 2017). An earlier study by Yaz et al. also showed the cytotoxic effect of clomifene on glioma cells *in vitro* (Yaz et al., 2004). Furthermore, mutant IDH1-mediated H3K9me3 levels were decreased in mouse xenografts after treatment of clomifene.

### Biguanides (metformin and phenformin)

Metformin is an oral drug belonging to the cationic biguanide class, which is a first-line medication in the treatment of Type II diabetes mellitus (TIIDM). It is a readily available, inexpensive, and safe drug (Molenaar et al., 2017). Phenformin is a lipophilic analog of metformin, which is also used in TIIDM management. However, phenformin was withdrawn from TIIDM treatment by the FDA and European Medicines Agency (EMA) in the 1970s because of its lactic acidosis side effect (Molenaar et al., 2017). Although the exact mechanism is unknown, metformin is believed to inhibit gluconeogenesis, increase glycolysis, and increase insulin sensitivity by promoting peripheral glucose uptake. Interestingly, metformin was correlated to cancer prevention and thus is of interest in drug repositioning for cancer treatment (Adeberg et al., 2015; Seliger et al., 2016).

Biguanides were shown to demonstrate anti-gliomagenic properties by inhibiting GBM cell proliferation, decreasing migration, inducing apoptosis, decreasing angiogenesis, reducing TMZ resistance, reducing self-renewal, and inhibiting stemness of GSCs (Ferla et al., 2012; Ucbek et al., 2014; Elmaci and Altinoz, 2016; Jiang et al., 2016; Yang et al., 2016).

Biguanides also induce tumor regression and prolong survival in xenograft models. Several mechanisms have been postulated as to why biguanides exhibit anti-tumor characteristics. Biguanides inhibit complex I of the electron transport chain in the mitochondria of cells and cause accumulation of AMP levels (Figure 1). This in turn upregulates LKB1-5′-AMP-activated protein kinase (LKB1-AMPK) and subsequently inhibits the mammalian target of rapamycin complex 1 (mTORC1) (Kast et al., 2011; Aldea et al., 2014). As mTORC1, a key signal for tumorigenesis, is reduced, cancer cell growth also decreases. Intracellular mitochondrial-dependent ATP production is switched to glycolytic ATP production with more lactate production (Sesen et al., 2015). AMPK activation also directly reduces insulin and insulin-like growth factor-1 (IGF-1), which normally stimulate cell growth (Elmaci and Altinoz, 2016). In addition, biguanides also inhibit AMPK and signal transducer and activator of transcription 3 (STAT3) pathways (Ferla et al., 2012).

Interestingly, biguanides modulate microRNAs (miRNAs) that regulate the posttranslational gene expression of cells (Jiang et al., 2016). These miRNAs are critical for energy metabolic pathways, cell cycle, and stemness. For instance, phenformin increases expression of miR-124 and let-7, which are essential for self-renewal of GSC (Jiang et al., 2016) (Figure 1). Biguanides can increase the bioavailability of let-7 when its binding partner H19 is downregulated, and thus enhancing the inhibitory effect of let-7 on the oncogene HMGA2 (Lee and Dutta, 2007; Jiang et al., 2016). Biguanides inhibit glutamate dehydrogenase and reduce glutaminolysis and the production of oncometabolite D-2-HG in IDH1/2 mutated glioma (Molenaar et al., 2017). Finally, Gritti et al. demonstrated that metformin can selectively target chloride intracellular channel-1 (CLIC1) in GBM and this inhibition leads to G1 arrest of GSCs. Importantly, the multiple pathways targeted by biguanides make them especially promising candidates for repurposing for the treatment of heterogeneous tumors such as GBM.

Similar to clomifene, metformin when combined with chloroquine can also reduce IDH1-mutated glioma tumors in clinical trials. *In vivo* studies have demonstrated that metformin and chloroquine can pass through the BBB appreciably. However, high levels of metformin efflux transporters have been reported in glioma and this raises concerns regarding the intratumoral bioavailability of metformin (Molenaar et al., 2017). Molenaar et al. thus proposed to use phenformin instead because phenformin is lipid-soluble and does not depend on transporters to enter cells.

### Repaglinide

Repaglinide is a non-sulfonylurea insulin secretagogue belonging to the family of meglitinide that was invented in 1983. It is an oral medication used in addition to diet and exercise to control postprandial glucose excursions for the treatment of TIIDM. It promotes the early release of insulin from the pancreas beta-islet cells by closing ATP-dependent potassium channels in the membrane of beta cells (Xiao et al., 2017). This results in depolarization and calcium influx to induce insulin secretion.

Repaglinide has been reported to kill hepatic, breast, and cervical carcinoma cells (Xiao et al., 2017). Xiao et al. first identified repaglinide as a potential candidate in GBM and verified that *in vitro* and *in vivo*. Other than its ability to inhibit proliferation and migration of GBM cells *in vitro*, GBM-bearing mice treated with repaglinide also survive longer (Xiao et al., 2017). The authors postulated the effects seen were via inducing apoptosis, repressing autophagy, or immune-activation. This was thought to be achieved via downregulation of the mitochondria-mediated anti-apoptotic protein Bcl-2, as well as engagement of the Beclin-1 and PD-1/PD-L1 immune pathway (Figure 1).

### Cyclin-dependent kinase inhibitors (CKIs)

Cyclin-dependent kinases (CDKs) are important checkpoint regulators in the cell cycle. In GBM cells as in most proliferating cells, CKIs arrest cells in S phase and at the G2/M transition. Importantly, CDK4/6 inhibitors have been approved for the treatment of breast cancer and thus there is considerable potential to repurpose this class of drugs (de Groot et al., 2017).

Flavopiridol, a first generation CKI, has cytotoxic effects on GBM cells (Cobanoglu et al., 2016). It also enhances the anti-tumorigenesis effect of TMZ in GBM cells by inhibiting DNA repair activity at the G2M transition. However, first generation CKIs are disfavored because of their low specificity. Roscovitine, milciclib, palbociclib, purvalanol A, and dinaciclib are some of the examples of second-generation CKIs developed later (Table 1). Jane et al. demonstrated that dinaciclib, which selectively inhibits CDK1, CDK2, CDK5, and CDK9, can reduce GBM cell proliferation independent of p53 status (Jane et al., 2016). Palblociclib, a CDK4/6 inhibitor, can also inhibit the cell cycle in GBM by inhibiting Rb1 phosphorylation (Lubanska and Porter, 2017). When combined with TMZ and radiotherapy, the brain penetrant CKIs abemaciclib and pablociclib show a GBM tumor suppressing effect. Although these CKIs show promising results in pre-clinical studies, most of them fail to make it into clinical trials, possibly due to their limited therapeutic window.

Interestingly, recent studies have looked into the non-canonical binding partners of CDKs which are usually not inhibited by CKIs. It is hypothesized that these non-canonical binding partners can continue activating CDKs in the presence of CKIs. Some of the binding partners described are Spy1A1, p35, and p39 protein (Lubanska and Porter, 2017). Lubanska and his team investigated the inhibition of Spy1A1 protein in brain tumor initiating cells as a potential target in GBM (Lubanska and Porter, 2017). Pre-clinical studies using BTICs have shown that Spy1 inhibition reduces cell proliferation and regulates stemness of cells (Lubanska et al., 2014).

### EGFR inhibitors

EGFR is a transmembrane glycoprotein with a molecular weight of 170 kDa. When EGFR is bound to a ligand, it can activate downstream pathways such as PI3K/Akt, mTOR, or Ras/Raf/MAPK, and stimulate GBM progression, angiogenesis, and invasion.

EGFR is overexpressed, amplified, or mutated in GBM (Clarke et al., 2013; Miranda et al., 2017). EGFR variant III (EGFRvIII), which is a truncated yet constitutively active form of EGFR, is present in 20–30% of glioblastoma tumors. This variant is the result of deletion of 267 amino acids in its extracellular domain. EGFRvIII is thought to stimulate proliferation of GBM by PKA-dependent phosphorylation of Dock180 (Miranda et al., 2017). However, multiple attempts to inhibit this pathway in glioblastoma using EGFR tyrosine-kinase inhibitors (TKIs) and naked monoclonal antibodies (mAbs) have not been successful. Some of the TKIs that are used in glioma studies are erlotinib and gefitinib; and the mAbs utilized are nimotuzumab and cetuximab. However, whether this is a pharmacodynamic failure (the drug is not effective against glioblastoma cells) or a pharmacokinetic failure (the drug is unable to achieve a therapeutic level in the tumor due to the BBB) is still unclear. Immunotoxins, a group of antibody-drug conjugates (ADCs) have been generated to overcome drug delivery issues. An antibody targeting EGFR is conjugated to a linker and a cytotoxic payload (this can be drug, bacterial toxin, or radioactive isotope). ADCs recognize cell surface receptors, get internalized into the cytoplasm, transported to lysosomes for degradation, and subsequently release their payload. Studies have shown that ADCs can deliver higher concentrations of drugs to the tumor tissues, as compared to systemic administration of drug alone. ADCs have been shown to be effective in inhibiting glioblastoma growth *in vivo*.

In the past 3 years, ADCs have been used in clinical trials for glioma, namely ABT-414 and AMG-595 (Gan et al., 2017). ABT-414 is an ADC that is comprised of an anti-EGFR antibody conjugated to monomethyl auristatin F (MMAF), an inhibitor of tubulin assembly. Although it only penetrates BBB partially, it is hypothesized that this ADC can overcome resistance of GBM cells. A Phase 1 study showed that ABT-414 is safe and pharmacokinetically acceptable in newly diagnosed and recurrent GBM patients. Although most patients developed ocular toxicity, they are treated with corticosteroids and respond well to this treatment (Gan et al., 2017). Therefore, ADC therapy is an exciting promising avenue for the treatment of GBM and other gliomas.

### Statins

HMG-CoA (β-hydroxy-β-methylglutaryl coenzyme A) reductase inhibitors, or statins, are the most widely used lipid-lowering agents in the clinic. Among the commonly used statins are lovastatin, pravastatin, rosuvastatin, and simvastatin. These inhibit the conversion of HMG-CoA to mevalonate, a cholesterol precursor, and reduce mortality of a large number of patients with cardiovascular diseases (Table 1). The reduction of mevalonate also reduces farnesyl pyrophosphate (FPP) or geranylgeranyl pyrophosphate (GGPP), and the subsequent post-translational isoprenylation of GTP-binding protein including Ras, Rac, and Rho. These proteins are important for cell proliferation and are often mutated or amplified in several cancers including glioma.

Gais et al. in 2014 and Bhavsar et al. in 2016 have epidemiologically studied the pleiotropic effect of pre-operative use of statins on the prognosis of GBM patients (Gaist et al., 2014; Bhavsar et al., 2016). Possibly due to the differences in study design, they have generated mixed results (Lu and McDonald, 2017). Nevertheless, several experimental studies have shown the considerable cytotoxic activities of statins in GBM in time- and dose-dependent manners (Yanae et al., 2011). Multiple mechanisms of these statins in inhibiting GBM are: 1. TNF-related apoptosis-inducing ligand (TRAIL)-sensitizing effect; 2. an increase in expression of pro-apoptotic protein Bim; 3. reduction of the MAPK-dependent pathway and GTPase activation; 4. suppression of extracellular regulated kinase 1/2 (ERK1/2) and Ras/PI3K/Akt pathway; and 5. activation of c-Jun N-terminal kinase 1/2 (JNK1/2) (Tapia-Perez et al., 2011; Yanae et al., 2011) (Figure 1). However, a higher dose of statins is usually needed to achieve a therapeutic benefit, which may be accompanied with increased toxicity.

## Targeting cell death pathways in glioblastoma

A major goal of repurposing FDA approved compounds for GBM is to identify drugs that are cytotoxic rather than cytostatic in GBM as the ultimate goal of therapy is to eliminate remaining cancer cells after standard treatment. However, the molecular pathways controlling cell death in GBM are not completely understood. Apoptosis is a well-studied programmed cell death (PCD) pathway (Valdes-Rives et al., 2017). This intricate, tightly regulated, cellular process is widely considered to be a fundamental component of numerous processes including turnover in normal cells. Many anti-GBM therapies take advantage of PCD pathways, for instance to induce apoptosis in GBM cells, by the employment of drug treatments, chemotherapeutic agents and radiotherapy strategies. Most of the drugs repurposed for GBM treatment as discussed earlier can induce apoptosis in GBM cells. Escape from apoptosis is also one of the hallmarks of carcinogenesis, including the progression of GBM (Wong, 2011).

One of the major causes for GBM tumor expansion is the inability of the treated cells to undergo apoptosis. Nonetheless many players of the apoptotic cascades are present in GBM cells and can be modulated therapeutically. Furthermore, if GBM cells can regain susceptibility to apoptosis through effective intervening therapies, a significant improvement in treatment success will be achieved. Further elucidating the oncogenic forces that are driving resistance to apoptosis and how to target them in GBM could provide interesting insights and open doors to future investigations on how to overcome cell death resistance.

Numerous components are part of apoptosis signaling but a family of conserved cysteine-dependent aspartate-directed proteases known as caspases, are the central module initiating and facilitating the apoptosis-signaling cascade (Elmore, 2007). There are two distinct functional groups of caspases that are essential for carrying out apoptosis: initiator caspases (caspase 2, 8, 9, and 10) and executioner caspases (caspase 3, 6, and 7) (Elmore, 2007). Initially, caspases are expressed in the inactive form, but are rapidly cleaved and sequentially activated in the presence of extrinsic death receptor activation and other intrinsic apoptotic stimuli (Elmore, 2007). Canonically, initiator caspases are subjected to auto-proteolytic cleavage whereas executioner caspases are cleaved by initiator caspases (Howley and Fearnhead, 2008). The cleavage and activation of initiator caspases results in the stimulation of the cleavage and activation of several executioner caspases. Executioner caspases have been implicated in the degradation of over 600 cellular components that are necessary to induce the morphological changes that underlie apoptosis (Sollberger et al., 2014). This highly ordered proteolysis allows for an amplifying cascade for the degradation of cellular components and a minimization of immune response during apoptosis.

Several apoptotic pathway components are dysregulated in GBM. Prominent examples of these include inactivating mutations or altered expression of specific proteins or their downstream signals. These include p53, inhibitor of apoptosis proteins (IAPs), and the B-cell lymphoma (BCL-2) family of proteins (Fels et al., 2000; Lytle et al., 2005; Nagpal et al., 2006; Ziegler et al., 2008; Berger et al., 2010; Liwak et al., 2013; Yang et al., 2014; Daniele et al., 2015; Wang et al., 2016).

The tumor suppressing gene, p53, plays a critical role in regulating the response mechanisms of DNA damage through apoptosis and cell cycle signaling. Likewise, p53 alteration has an effect in a vast number of cancers, including GBM (Nagpal et al., 2006). p53 is regulated by murine double minute (MDM) 2 and 4, which inhibit p53 stability or activity. Following DNA damage, p53 is activated and induces the transcription of response genes such as p21^Cip1^, a negative regulator of the cell cycle, or BCL-2-like protein 4 (BAX), a mediator of apoptosis (Essmann and Schulze-Osthoff, 2012). In addition to its regulation through transcriptional activity, p53 also can promote apoptosis through transcription-independent mechanisms and direct interactions with members of the BCL-2 and the caspase family of proteins (Essmann and Schulze-Osthoff, 2012). In a comprehensive genomic study by The Cancer Genome Atlas (TCGA) Research Network, human GBM genes and associated pathways were characterized. This study revealed that p53 signaling was altered in 87% of all GBM patients by mutations in at least one component of the pathway (Biasoli et al., 2014). Some examples of drugs that target p53 reactivation are Nutlins, benzodiazepinediones, spiro-oxindoles, RITA (Reactivation of p53 and induction of tumor cell apoptosis), and Serdemetan (Yu et al., 2014). Given the importance of the p53 pathway in the regulation of apoptosis in human GBM and many other cancers, several efforts have been made to develop both pharmacological and biological therapeutics targeting this pathway. However, due to delivery, selectivity, and toxicity problems, many of these therapies fail in development and clinical trials. Hence, repurposing FDA approved drugs that target p53 directly or indirectly for use in GBM treatment may have therapeutic potential.

IAPs are cellular checkpoints that can inhibit pro-apoptotic caspase signaling. Additionally, IAPs have been found to modulate cell invasion and metastasis in GBM and several cancers. All IAPs contain a Baculovirus Inhibitor of apoptosis protein Repeat (BIR) domain and are commonly termed BIRCs. The IAP family consists of six primary members: NLR family Apoptosis Inhibitory Protein (NAIP or BIRC1), Cellular IAP (CIAP 1 and 2 or BIRC2 and 3), X-linked IAP (XIAP or BIRC4), Survivin (BIRC5), and *BIR* repeat-containing ubiquitin-conjugating enzyme (BRUCE or BIRC6) (Hunter et al., 2007; Owens et al., 2013). BIRC4 is the only IAP that directly associates with caspases. It has been shown to bind with high affinity to executioner caspase 3 and 7, in addition to initiator caspase 9, in turn inhibiting apoptotic function. All other IAPs do not bind to caspases directly. A leading model suggests that apoptosis inhibition is achieved by forming complexes with other partners such as BIRC4 (Silke and Meier, 2013). Several compounds have been shown to cause degradation of IAPs including the Smac mimetic, SM-164, small molecule inhibitors of BIRC4, Arylsulfonamides and Embelin, and a small molecule inhibitor of BIRC5, YM155 (Owens et al., 2013). A comprehensive list of IAP inhibitors can be found in Owens et al. (2013). Inhibition of IAPs has been implicated in both inducing apoptosis directly but also sensitizing cells to radiation and chemotherapy treatment (Rathore et al., 2017). Repurposing drugs that target this family of proteins may be a promising strategy in treating GBM but may also have synergistic potential when used in combination with other treatments.

The BCL-2 protein family has a key role in tightly regulating the mitochondrial pathway of apoptosis. Members in this family functionally promote or inhibit apoptosis. All proteins in this family share at least one of four BCL-2 homology (BH) domains (Wang et al., 1996). The proteins that have anti-apoptotic functions contain both BH1 and BH2 domains (BCL-2 and BCL-X) while the proteins that exhibit pro-apoptotic functions widely lack sequence homology to the family, but contain the BH3 domain only (BAX, BAK, BID, BAD) (Wang et al., 1996). Anti-apoptotic signaling is achieved either by sequestering caspases or by preventing the release of mitochondrial apoptosis driving factors that activate caspases, such as cytochrome c and apoptosis-inducing factor (AIF), into the cytoplasm. By contrast, pro-apoptotic BCL-2 members, trigger the release of caspases from death antagonists and act on the mitochondrial permeability transition pore to induce the release of pro-apoptotic mitochondrial factors into the cytoplasm. In patients with GBM, expression of anti-apoptotic BCL-2 proteins is increased, which may contribute to apoptotic resistance and relapse that is commonly observed (Fels et al., 2000; Martin et al., 2001). A few examples of drugs that inhibit BCL-2 family of proteins that have been used in cancer treatment are Venetoclax, Servier-1, and Disarib. Regulating the homeostasis of anti and pro-apoptotic BCL-2 family proteins may be a worthwhile strategy in combatting GBM. Repurposing drugs that target activation of BH3 domain only BCL-2 proteins could be used to induce apoptosis in GBM. Importantly, repositioning drugs inhibiting the function of anti-apoptotic BCL-2 proteins could restore sensitivity of some apoptotic-inducing treatments of GBM.

Necroptosis is a caspase-independent, pro-inflammatory form of PCD that can also be pharamacologically targeted in GBM (Jiang et al., 2011). Morphologically, necroptosis shares similar features to necrosis such as loss of plasma membrane integrity. However, cellular membrane permeabilization induced by necroptosis signaling is tightly regulated. Necroptosis induction begins with activation of the tumor necrosis factor (TNF) family of cytokines or TRAIL (Vanden Berghe et al., 2010). These stimuli are known to also regulate cell survival and apoptosis induction. The activated receptor then interacts with Receptor-interacting serine/threonine-protein kinase (RIPK) 1 and recruits IAPs such as BIRC2 and 3 (Christofferson and Yuan, 2010). This results in the formation of a membrane associated complex that leads to cell survival through NF-κB and mitogen-activated protein kinases (MAPKs) pathways. When IAPs are inhibited, RIPK1 is rapidly deubiquinated by cylindromatosis lysine 63 deubiquinase (CYLD) and disassociated from the membrane bound complex (Christofferson and Yuan, 2010). The free RIPK1 binds to the adaptor protein Fas-associated protein with death domain (FADD) and caspase 8, which in turn activates caspase 8 and induces apoptosis. In addition, active caspase 8 dynamically inhibits necroptosis by cleaving its core regulators, RIPK1 and RIPK3. In the event that caspase activity is inhibited, necroptosis is executed. RIPK1 binds with RIPK3 to form an insoluble amyloid complex known as the necrosome. The formation of the necrosome promotes autophosphorylation of RIPK3, which then recruits and phosphorylates the pseudo-kinase, mixed lineage like kinase (MLKL). Phosphorylated MLKL oligomerizes and is inserted in to the membrane to form a pore, leading to necroptosis by the loss of plasma and intracellular membrane integrity (Christofferson and Yuan, 2010; Vanden Berghe et al., 2010; Geng et al., 2017). Necroptosis initiation is often viewed as a backup mode to ensure cell death execution, but emerging evidence suggest that necroptosis may act as a primary cell death mode under certain pathological conditions. Recent reports suggest that under conditions where apoptosis is inhibited, apoptosis inducing drugs, such as IAP and BCL-2 inhibitors, can induce necroptosis in cancer cells (Su et al., 2016). Targeting this novel cell death pathway may have therapeutic potential in apoptosis-resistant GBM cells (Jiang et al., 2011). Repurposing drugs that activate necroptosis may not only be used as an effective primary treatment but could also be used in combination with other therapies after drug-resistance develops.

Autophagy is a catabolic process in which cells induce lysosomal degradation of cellular components. It is a highly-conserved pathway that has a pivotal role in cell stress response such as nutrient starvation, DNA damage, and organelle damage (Glick et al., 2010). Autophagy is regulated primarily by a large number of proteins, from a family identified in yeast known as autophagy related genes (ATGs) (Glick et al., 2010). mTOR signaling is also a central regulator of autophagy (Dunlop and Tee, 2014). Activation of mTOR by AKT and MAPK signaling suppresses autophagy signaling, while mTOR inhibition by the negative regulators AMPK and p53 drives the process forward (Jung et al., 2010). Mammalian kinase orthologs of ATG1, UNC-51-like kinase (ULK) 1, 2, and 3 initiate autophagy downstream of mTOR. ULK 1 and 2 form a large complex with mATG13, FIP200, and a PI3K Class III complex, which contains the proteins Beclin-1, hVps34, p150, and ATG14 (Itakura and Mizushima, 2010). This complex eventually promotes invagination of the membrane, which leads to the formation of the autophagosome and subsequent execution of autophagy by fusion of the autophagosome with the lysosome by the Atg5–Atg12 and the microtubule-associated protein 1 light chain 3 (LC3) pathway (Jiang and Mizushima, 2014).

Autophagy has garnered much attention in the cancer field and is widely being evaluated for its potential in GBM therapy. Lefranc et al. demonstrated that glioma therapies are more likely to be successful by inducing autophagy rather than apoptosis, as two potent cytotoxic drugs, TMZ and rapamycin, induce autophagy (Lefranc and Kiss, 2006). Furthermore, several reports indicated that Atg5 and LC3 loss of function promotes glioblastoma progression (Lefranc and Kiss, 2006). Autophagy certainly plays a role in inhibiting tumor growth progression and metastasis. Yet the role of autophagy causing cell death directly in contrast to occurring in parallel to PCD needs to be further elucidated. It has been shown to function as a tumor suppressor as well as to play a role in tumor cell survival (Yonekawa and Thorburn, 2013). It is important to note that cells undergoing autophagy are found in high numbers under certain conditions such as nutrient starvation induced PCD, but autophagy in this context is not considered to be a PCD pathway because inhibition of autophagy attenuates cell death rather than inhibiting it (Tsujimoto and Shimizu, 2005; Gozuacik and Kimchi, 2007). In parallel, it has been shown that hyper-activation of autophagy can indeed lead to PCD that is morphologically distinct from other PCD pathways, termed autophagic cell death (Tsujimoto and Shimizu, 2005). Autophagy and apoptotic pathways substantially interact; the two processes both negatively and positively regulate each other (Ryter et al., 2014). For example, many apoptosis inducing signals, such as TNF, TRAIL, and FADD, also induce autophagy (Das et al., 2012).

Therefore, identifying FDA approved compounds that modulate these components in GBM may be especially attractive since this will increase the chances of eliminating GBM cells within tumor cells.

## Computational and data-driven approaches to identify drugs with efficacy in glioblastoma

Various computational and data-driven methods are applicable for the systematic identification and prioritization of candidate drugs for repurposing in glioblastoma. Such *in-silico* methods can be based on known GBM targets or pathways or entirely data-driven without requiring knowledge on the mechanism of action. In the former case, the goal is to identify drugs that target specific pathways or proteins relevant in GBM or, inversely, identify such targets among approved drugs. Computational target prediction can broadly be classified into ligand- and structure-based methods. Ligand-based *in silico* target screening aims to predict biological targets based on the chemical structure of the drug (Jenkins et al., 2007). This approach leverages large bioactivity databases including public resources such as ChEMBL (Bento et al., 2014) and PubChem (Wang et al., 2017a) or licensed databases such as the KKB (Sharma et al., 2016) in combination with machine learning (Nidhi et al., 2006; Schurer and Muskal, 2013; Bento et al., 2014) or statistical scoring (Keiser et al., 2009). As in many other areas deep learning has recently received a lot of attention (Gawehn et al., 2016). Structure-based approaches make use of the ever-increasing corpus of experimentally determined protein structures or computational protein structure models using docking (Lauro et al., 2011) or binding site similarity predictions at the genome-wide scale (Hwang et al., 2017). Cheminformatics ligand- and structure-based repositioning approaches are well established and have also been extensively reviewed for drug repositioning, for example recently by March-Vila et al. (2017). Importantly, several well annotated databases of approved drugs and compounds in clinical trials are publicly available including ChEMBL (Bento et al., 2014), DrugBank (Wishart et al., 2006), and DrugCentral (Ursu et al., 2017).

In contrast to mechanism- or target-based drug repurposing, system-wide perturbation response signatures can be used to identify repurposing opportunities without bias for specific targets or pathways. One of the most systematic and comprehensive of such data-driven approaches is the Connectivity Map (CMap) project, which is based on gene expression signatures (Lamb et al., 2006). Initially covering 164 drugs and a few cell lines, it has recently been scaled by more than 1,000-fold using the L1000 reduced representation high throughput gene expression profiling platform (Subramanian et al., 2017). These data have been generated as part of the Library of Integrated Network-based Cellular Signatures (LINCS) project along with several other cellular perturbation response signatures and computational tools (Keenan et al., 2017). LINCS has been developing a larger-scale integrated approach to data curation, with baseline gene expression signatures for 99 cell lines and transcriptional profile responses to over 30,000 perturbations and many other datasets. To ensure validity and consistency, as well as to allow comparison across cell lines and perturbations for different datasets, the data in the LINCS database were collected and processed in a highly standardized and coordinated manner enabling integrated analysis of drug action (Vempati et al., 2014; Vidovic et al., 2014). LINCS datasets are available in different data-type specific repositories and via the LINCS Data Portal (Koleti et al., 2017).

Using LINCS data in a recent study, the ability to predict novel drug repositioning candidates based on several perturbational features was assessed in four cancer types, including glioblastoma. Reference expression profiles for GBM tissue and controls were downloaded from TCGA and a list of known drugs in glioblastoma was compiled from public databases. For these compounds, different signature types were generated based on chemical structure, targets, and gene-expression data. Classifiers were constructed for each signature type as well as combinations and evaluated by cross validation, comparison against data NCI-60 data, and cell viability screening. The gene expression-based signatures gave the best predictions for anti-cancer hits (Lee H. et al., 2016). This study highlights the utility of data-driven approaches to identify potential drugs based on transcriptional responses to drug treatments.

To address the considerable variability in efficacy of targeted cancer drugs for individual patients, Armetov et al. used gene expression signatures of individual tumor samples and predicted a drug score based on signaling pathway activation analysis (OncoFinder algorithm). They tested the approach for five drugs in seven cancer types and reported significant correlation of responders to drug treatment and the percent of tumors showing high drug scores (Artemov et al., 2015).

Beyond the use of individual approaches for drug repositioning, such as gene expression, target predictions, or pathway analyses, integrative “multi-scale” methods in computational pharmacology that integrate multiple resources and data types can enable the discovery of novel associations of drugs and diseases; such data types can include drug target interaction data, gene expression data, phenotypic drug screening data, drug side effects, and electronic health records (Hodos et al., 2016).

Recently, researchers at the Broad Institute have established the Drug Repurposing Hub, which is a database of 4,707 compounds, including 1,988 launched drugs and 1,348 compounds that have reached clinical trial Phase 1–3 (Corsello et al., 2017). It is a highly curated resource, integrating various information including detailed target annotations, mode of action, disease indications, and commercial availability for a very comprehensive list of approved drugs and clinical compounds. The compounds were carefully curated and annotated including chemical structures, purity, mechanisms of action based on several databases and literature curation, their approved indications and clinical trial status, as well as supplier IDs for commercial sources. A well designed graphical user interface allows querying this information (https://clue.io/repurposing).

The interactions among fundamental molecular entities and processes involving genes, transcripts, proteins, metabolites are tightly regulated to sustain a healthy biological system. In a diseased state, this normally interconnected network is disrupted by stressors and the aim of the therapy is to restore the system to its normal state. As discussed in Lee et al., gene expression data can be an excellent predictor of drug repositioning efficacy (Lee H. et al., 2016). As sequencing techniques improve and the availability of genomic data increases, combined transcriptomes and multi-scale integrative methods in computational pharmacology improve predictions of drug-disease associations and enable computational drugs repurposing. One integrated effort to relate genes to diseases is the Illuminating the Druggable Genome (IDG) project (https://commonfund.nih.gov/idg/). A specific priority for IDG is to identify understudied disease relevant targets. All IDG data and a query interface are available via the Pharos Data Portal (Nguyen et al., 2017).

Although these databases are important resources for identifying single repurposed compounds in GBM, it is likely essential to identify combinations of drugs for the treatment of GBM. Advances in single cell sequencing allow the characterization of sub-populations in individual GBM tumors based on gene expression to identify small molecules that have the greatest probability of affecting pathways common in all malignant cells within a tumor (Patel et al., 2014). In addition to intratumoral heterogeneity in GBM, there is also intertumoral heterogeneity. Combinations of drugs with different mechanisms of action are one approach to increase the success rate in drug repurposing (Sun et al., 2016). This is particularly relevant in heterogeneous cancers where clinical duration is often limited due to emerging resistance. Computational approaches as described above can be used to prioritize combination therapies; either rationally based on known mechanism of action or data-driven, for example using gene expression signatures. With the knowledge of targets, it is also possible to rationally design single-agent poly-pharmacology compounds (Allen et al., 2015).

To help identify patient-specific therapies we have previously described a bioinformatics pipeline to identify genes and pathways that are dysregulated in a particular GBM tumor (Stathias et al., 2014). This was accomplished by first performing RNA-sequencing of each GBM tumor and then using the significant sequencing information in TCGA to increase statistical power of the analyses. We found that by using both a hypergeometric test and Pearson correlation, we can identify networks dysregulated in each GBM tumor. Importantly, we are currently integrating this approach with the LINCS perturbation response gene expression signatures to identify therapeutic combinations in GBM and other gliomas. Although these computational approaches have not been validated in a clinical setting, it is our ultimate hope that our *in vitro* and *in vivo* studies will lead to patient specific drug combinations for the treatment of GBM.

## Conclusion

GBM is a deadly primary parenchymal central nervous system neoplasm disease with very dismal prognosis. By bypassing time-consuming chemical optimization and toxicology testing in drug development steps, repositioning of existing FDA-approved drugs can help to better manage GBM. As compounds need to be brain penetrant to have maximum efficacy in GBM, it is ideal to select compounds that are known to pass the blood brain barrier and are not substrates of efflux transporters. Many antipsychotic compounds are known to cross the blood brain barrier and can be directly utilized for treating GBM if they are shown to be effective in reducing GBM growth *in vivo*. In addition, non-antipsychotic compounds can also be utilized after demonstrating efficacy in GBM animal models and robust brain penetrance. Thousands of compounds have been approved for human use or are in advanced clinical testing. In addition to the rational selection of antipsychotics and non-antipsychotic drugs with likely efficacy in GBM, advanced computational tools are now available to prioritize prospective drugs and drug combinations for GBM. We propose that uncovering pathways controlled by each drug is essential for identifying therapeutic combinations for treating GBM. Identifying such combinations is essential for treating GBM tumors that exhibit both intertumor and intratumor heterogeneity. Future studies are needed to identify drug sensitive cell types within tumors without affecting normal brain cells.

## Author contributions

SKT and NGA conceived the concept and idea of the present review. SKT and NGA designed the strategy and selected the topics to be discussed. SKT did literature searches, read the references, and wrote the first outline of the review. AJ and AKM reviewed and contributed a section into final manuscript. CBN, SCS, and NGA reviewed the manuscript and helped with the final writing. The content of figures was conceived and prepared by SKT and NGA.

### Conflict of interest statement

CBN has been funded by National Institutes of Health (NIH), Stanley Medical Research Institute. For last three years, he has consulted for Xhale, Takeda, Taisho Pharmaceutical Inc., Prismic Pharmaceuticals, Bracket (Clintara), Total Pain Solutions (TPS), Gerson Lehrman Group (GLG) Healthcare & Biomedical Council, Fortress Biotech, Sunovion Pharmaceuticals Inc., Sumitomo Dainippon Pharma, Janssen Research & Development LLC, Magstim, Inc., Navitor Pharmaceuticals, Inc., TC MSO, Inc., Intra-Cellular Therapies, Inc. He is also a stockholder in Xhale, Celgene, Seattle Genetics, Abbvie, OPKO Health, Inc., Network Life Sciences Inc., Antares, BI Gen Holdings, Inc. He serves as member of the scientific advisory boards for American Foundation for Suicide Prevention (AFSP), Brain and Behavior Research Foundation (BBRF) [formerly named National Alliance for Research on Schizophrenia and Depression (NARSAD)], Xhale, Anxiety Disorders Association of America (ADAA), Skyland Trail, Bracket (Clintara), RiverMend Health LLC, Laureate Institute for Brain Research, Inc. He is a member of Board of Directors for AFSP, Gratitude America, ADAA. His Income sources or equity of $10,000 or more are from American Psychiatric Publishing, Xhale, Bracket (Clintara), CME Outfitters, Takeda. He holds the patents for: Method and devices for transdermal delivery of lithium (US 6,375,990B1); Method of assessing antidepressant drug therapy via transport inhibition of monoamine neurotransmitters by *ex vivo* assay (US 7,148,027B2). SKT, AJ, AKM, and SCS declare that the research was conducted in the absence of any commercial or financial relationships that could be construed as a potential conflict of interest. NGA is a shareholder of Epigenetix, Inc.
